# Multiple lipid binding sites determine the affinity of PH domains for phosphoinositide-containing membranes

**DOI:** 10.1126/sciadv.aay5736

**Published:** 2020-02-19

**Authors:** Eiji Yamamoto, Jan Domański, Fiona B. Naughton, Robert B. Best, Antreas C. Kalli, Phillip J. Stansfeld, Mark S. P. Sansom

**Affiliations:** 1 Department of System Design Engineering, Keio University, Yokohama, Kanagawa 223-8522, Japan.; 2 Department of Biochemistry, University of Oxford, South Parks Road, Oxford OX1 3QU, UK.; 3 Laboratory of Chemical Physics, National Institute of Diabetes and Digestive and Kidney Diseases, National Institutes of Health, Bethesda, MD 20892-0520, USA.; 4 Department of Physics, Arizona State University, Tempe, AZ 85287-1504, USA.; 5 Leeds Institute of Cardiovascular and Metabolic Medicine and Astbury Center for Structural Molecular Biology, University of Leeds, Leeds, UK.

## Abstract

Association of peripheral proteins with lipid bilayers regulates membrane signaling and dynamics. Pleckstrin homology (PH) domains bind to phosphatidylinositol phosphate (PIP) molecules in membranes. The effects of local PIP enrichment on the interaction of PH domains with membranes is unclear. Molecular dynamics simulations allow estimation of the binding energy of GRP1 PH domain to PIP_3_-containing membranes. The free energy of interaction of the PH domain with more than two PIP_3_ molecules is comparable to experimental values, suggesting that PH domain binding involves local clustering of PIP molecules within membranes. We describe a mechanism of PH binding proceeding via an encounter state to two bound states which differ in the orientation of the protein relative to the membrane, these orientations depending on the local PIP concentration. These results suggest that nanoscale clustering of PIP molecules can control the strength and orientation of PH domain interaction in a concentration-dependent manner.

## Teaser

Molecular simulations reveal that multiple lipid interactions are needed for high-affinity binding of protein domains to membranes.

## INTRODUCTION

Cell signaling and trafficking are regulated by peripheral membrane proteins that associate with cell membrane surfaces in a lipid-dependent fashion (*1*, *2*). Recognition of cell membranes is brought about by lipid recognition domains (*3*). The pleckstrin homology (PH) domains form a large and well-characterized family present in many membrane recognition proteins including, e.g., AKT and Btk (*4*, *5*). PH domains can bind to phosphatidylinositol phosphates (PIPs), which confer a molecular identity to the different membranes with a eukaryotic cell (*1*). Lipid cooperativity (i.e., interaction with more than one lipid molecule and/or species) may also play a key role in the recruitment of PH domains to cell membranes (*6*, *7*). Furthermore, it is thought that nanoscale lipid clustering may play a key role in the interactions of membrane proteins with lipids (*8*, *9*), in turn influencing the avidity of recognition proteins for membranes (*10*–*13*).

**Fig. 1:**
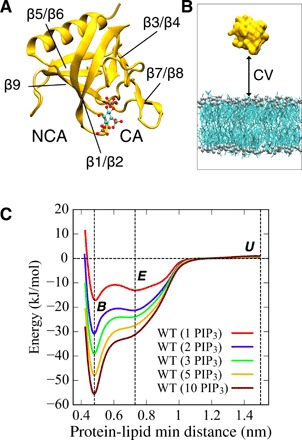
MD simulations for calculating PMFs of PH/PIP_3_ interactions. (**A**) Structure of the GRP1 PH domain (Protein Data Bank ID: 1FGY; yellow cartoon format) with an Ins(1,3,4,5)P_4_ molecule (ball and stick format) bound at the canonical site (CA). The approximate location of the noncanonical site (NCA) is indicated. (**B**) Collective variable (CV) based on the minimum distance between the protein and lipid as used in the REUS-MD simulations. (**C**) Potentials of mean force (PMFs) for the GRP1 PH domain interacting with lipid bilayers containing from 1 to 10 PIP_3_ molecules, showing the free energy of interaction as a function of protein-lipid minimum distance. The three vertical dashed lines correspond to the protein-lipid minimum distances of 0.48, 0.73, and 1.5 nm (see text for details).

The structure of a PH domain consists of ~120 amino acid residues folded into an antiparallel β sheet, followed by one or two amphipathic helices. Many structures of PH domains are known, a number of which include a PIP headgroup (i.e., an inositol phosphate) bound at a canonical binding site (CA) formed by positively charged residues of the β1/β2 and β3/β4 loops (see Fig. 1A) (*14*, *15*). A KXn(K/R)XR sequence motif in the β1/β2 loop determines contacts of the PH domain with different classes of PIP molecules. Certain PH domains, e.g., those of β-spectrin and ArhGAP9 (*15*, *16*), lack this motif and instead have an alternative, noncanonical binding site (NCA) on the opposite face of the β1/β2 loop in between the β1/β2 and β5/β6 loops. The crystal structure of the ASAP1 PH domain reveals two bound PIP headgroups, one at the CA and one at the NCA (*6*). This suggests that recruitment of PH domains to cellular membranes may involve binding to multiple PIP molecules by one domain.

Biophysical and computational studies have explored, in some detail, the mechanism of membrane binding by PH domains (*17*–*20*). A number of key aspects remain unresolved, in particular, the impact of PIP clustering on PH domain interactions with membranes and the influence on the mechanism and strength of binding of the presence of both canonical and alternative binding sites on the same PH domain. PIP concentrations in cell membranes are relatively low: less than 5% in the plasma membranes and about 10% in Golgi membranes (*21*, *22*). However, a number of studies have indicated that PIP molecules can cluster in cell membranes to form nanoscale domains, which, in turn, enhance interactions with proteins (*8*, *9*). In vitro studies have explored the effects of other phospholipids (*11*) and of Ca^2+^ ions (*10*) on PIP clustering and conformation. Both experimental and computational investigations have suggested clustering of PIP molecules around PH domains (*23*–*26*). The association of PH domains with cell membranes is influenced by sites distinct from the canonical PIP binding pocket (*27*), and a number of PH domains [e.g., that from ASAP1 (*6*)] have revealed multiple PIP-binding sites in crystal structures. Some PH domains have been demonstrated to bind cooperatively to PIPs [as indicated by, e.g., sigmoidal PIP dependence of binding to vesicles (*6*)]. It has also been shown that other anionic lipid species [e.g., phosphatidylserine (PS)] may contribute to the binding of PH domains to liposomes in a microarray-based assay (*7*) and that the presence of PS leads to a ~10-fold increase in the affinity of GRP1 PH for PIP_3_ in a bilayer (*17*). Analysis of 33 different yeast PH domains revealed that only 1 bound phosphoinositides with high affinity, while 6 other PH domains bound with moderate affinity and low specificity (*28*). Together, these data indicate that while many PH domains may have a relatively low canonical binding site affinity for PIPs, interaction of multiple PIP molecules and/or other anionic lipid species may enable overall tight binding of a PH domain to a membrane. Thus, PH domains may act via coincidence sensing, i.e., detection of (local) clusters of PIP molecules and/or other anionic lipids (*2*, *7*). Furthermore, binding of PH domains and other lipid-binding modules to membranes may, in turn, mediate PIP clustering by modifying the local lipid environment (*29*). PIP clustering affects the diffusivity of PH domains on the membrane surfaces, which is likely to play a role in regulating the function of membrane-bound proteins (*30*).

Molecular dynamics (MD) simulations enable investigation of both protein-lipid interactions (*31*) and the larger-scale organization of complex cell membranes (*32*). Simulations of PH domains have been used to explore the structure (*18*, *33*), dynamic mechanisms (*20*, *24*, *26*, *30*), and energetics (*34*, *35*) of PH domain/membrane interactions. Here, we exploit recent advances in replica-exchange umbrella sampling (REUS) of protein-lipid interactions (*36*) to explore how the binding free energy of the GRP1 PH domain changes with respect to the number of PIP_3_ molecules with which it interacts within a membrane. Comparison of our results with experimental estimates of the dissociation constant of PH domain from a PIP_3_-containing bilayer suggests that at least three PIP_3_ molecules interact with the PH domain. Our simulations also suggest a three-step mechanism for tight association of the PH domain with the membrane. These results have implications more generally for coincidence-sensing mechanisms of recognition of complex cell membranes by proteins containing PH domains.

## RESULTS

### Potentials of mean force for GRP1 PH/PIP_3_ interactions

To estimate potentials of mean force (PMFs) for the interaction of the GRP1 PH domain with PIP-containing lipid bilayers, we performed REUS-MD simulations (*37*) using a Martini coarse-grained (CG) model (*38*). REUS enables faster convergence relative to standard umbrella sampling (US) and also allowed us to avoid the need for a constraint on the PIP_3_ lipid head group as used in our previous PMF calculations (*34*). We used a collective variable (CV) based on minimum distance between the protein and lipid (see Methods for further details). We were able to extensively explore the free energy landscape (see below) of PH/membrane interactions, as the REUS approach allowed us to sample multiple binding and dissociation events, thereby revealing a potential binding pathway for the protein.

For the GRP1 PH domain, simulation systems with different concentrations of PIP_3_ in lipid bilayers were performed, i.e., with from 1 to 10 PIP_3_ molecules in each leaflet of the bilayer, corresponding to concentrations from 0.8 to 8%. As an initial configuration of the system, the PH domain was displaced away from the membrane surface. Simulations were performed for 15 μs for each replica, yielding a total REUS-MD simulation time of 240 μs and thus a total MD simulation time of over 1 ms for all of the systems explored.

PMFs as a function of the GRP1-PIP_3_ PH protein-lipid minimum distance are shown in Fig. 1C for different numbers of PIP_3_ molecules within the membrane. For a single PIP_3_ molecule in the protein-exposed leaflet, a major and a minor minimum are seen, with an overall minimum interaction energy of −17 kJ/mol relative to the unbound (*U*) state, in agreement with previous estimates (*34*). The two minima at minimum distances of 0.73 and 0.48 nm thus correspond to a loosely interacting state (subsequently to as the encounter state *E*; see below) and a more tightly bound state (*B*) of the PH domain on the membrane surface. As the PIP_3_ concentration is increased, the bound state *B* is increasingly stabilized relative to the encounter state *E* such that the free energy difference between these two states is 4 kJ/mol for the 1 PIP_3_ system, increasing to 20 kJ/mol for the 5 PIP_3_ system.

### Free energy landscapes for interaction

**Fig. 2:**
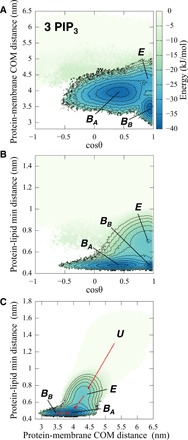
Free energy surfaces of the GRP1 PH domain interacting with a lipid bilayer including 3 PIP_3_ molecules in each leaflet. *E*, *B_A_*, and *B_B_* refer to the *Encounter*, *Bound_A_* and *Bound_B_* states of the PH domain when interacting with the membrane (see main text and Fig. 5B for further details). Three different projections of the free energy landscape are shown: (**A**) as a function of cosθ (where θ is the angle between a vector corresponding to the PH domain α helix and the *z* axis perpendicular to the membrane) and the protein-membrane COM distance, (**B**) as a function of cosθ and the protein-membrane minimum distance, and (**C**) as a function of the protein-membrane COM distance and the corresponding minimum distance.

To investigate the binding mechanism in more detail, exploring the orientation of the PH domain relative to the membrane surface, two-dimensional free energy surfaces were calculated for the system with three PIP_3_ molecules in each leaflet of the membrane. Thus, free energy surfaces (Fig. 2) were calculated as a function of (i) the protein-membrane center of mass (COM) distance versus cosθ (where θ is a tilt angle of the PH domain α helix relative to the membrane), (ii) the protein-lipid minimum distance versus cosθ, and (iii) protein-membrane COM distance versus the protein-lipid minimum distance.

From these free energy surfaces, it is evident that there are actually three states of the PH domain interacting with the membrane. The *Encounter* state (*E*) is characterized by a minimum distance of 0.73 nm and a COM distance of 4.3 nm. The bound state, characterized by a minimum distance of 0.48 nm, can be seen to be split into two orientational states *Bound_A_* (*B_A_*) and *Bound_B_* (*B_B_*), which differ in their COM distance such that *B_B_* is closer to the center of the bilayer. From the projection of the free energy landscape in Fig. 2C, we would suggest that the mechanism of binding is

$$U\to E\to B_{A}↔B_{B}$$

where the *B_A_* and *B_B_* states are of comparable stability.

**Fig. 3:**
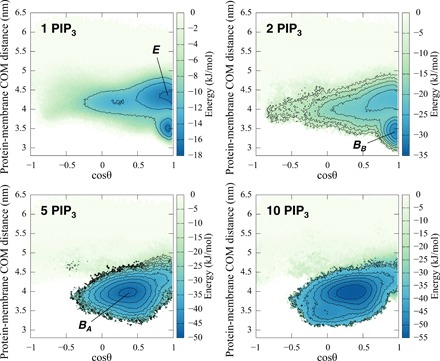
Free energy surfaces of the GRP1 PH domain interacting with a lipid bilayer including 1 to 10 PIP_3_ molecules in each leaflet. The free energy landscapes are shown as a function of cosθ and the protein-membrane COM distance.

Examination of these maps reveals how the free energy landscape changes depending on the concentration of PIP_3_ within the membrane (see Fig. 3). For the membrane containing one PIP_3_ molecule in each leaflet, states *E* and *B_B_* are of similar stability. For the 2 PIP_3_ system, the *B_B_* state becomes more stable, whereas as seen above for the 3 PIP_3_ membrane, the free energy of states *B_A_* and *B_B_* are about the same, and finally for 5 PIP_3_ only the *B_A_* state is seen. Thus, the concentration of PIP_3_ in the bilayer can alter not only the overall strength of interaction of the PH domain with the membrane but also the orientation of the bound protein relative to the bilayer.

### PH bound to multiple PIP_3_ molecules

**Fig. 4:**
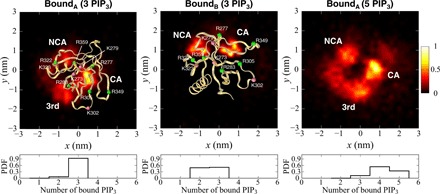
Clustering of lipids in the bilayer (*xy*) plane underneath a bound GRP1 PH domain. The density (unbiased density normalized by the maximum density) of phosphate headgroups of PIP_3_ molecules in the bilayer plane corresponding to each bound state is shown on a heat map scale from dark red to yellow. Peaks in the density corresponding to interactions with the canonical binding site (CA), the noncanonical binding site (NCA), and a third site (3rd; see main text for details) are shown. Density maps are shown for the PH domain in the *Bound_B_* and *Bound_A_* configurations interacting with a lipid bilayer including 3 PIP_3_ molecules in each leaflet and in the *Bound_A_* configuration interacting with a lipid bilayer including 5 PIP_3_ molecules in each leaflet (in this latter case, the protein is not shown in the interests of clarity). Bottom: Probability density functions (PDFs) for different numbers of PIP_3_ molecules bound to the PH domain in each of the states.

From the free energy surfaces, it is evident that there are two bound states of the PH domain, both with a protein-lipid minimum distance of 0.48 nm. Although the free energies of states *B_A_* and *B_B_* for the 3 PIP_3_ system are almost the same, they differ in their orientations relative to the bilayer and their interactions with PIP_3_ molecules in the membrane. In both states, the β1/β2 loop interacts with PIP_3_ headgroups at the membrane surface. In state *B_A_*, the α helix is away from the membrane surface, whereas in state *B_B_*, the β1/β2 loop interacts more closely with the membrane, and β3/β4, β5/β6, β9, and the α helix also approach the membrane more closely. From the probability densities of PIP_3_ molecules in contact with the PH domain, it can be seen that this shifts from two to three molecules bound in the 3 PIP_3_ system to four to five molecules bound in the 5 PIP_3_ system (Fig. 4). Examining the probability densities of PIP_3_ headgroups in the membrane plane in the vicinity of the bound PH domain, it can be seen that for, e.g., the 3 PIP_3_ simulation, in state *B_A_*, there are three regions of high PIP_3_ density, corresponding to the CA (Fig. 4), the NCA, and a third region adjacent to CA. In state *B_B_*, which penetrates more deeply into the bilayer, PIP_3_ molecules are largely restricted to the CA and NCA sites, with a higher density in the NCA region than for *B_A_*. In the presence of five PIP_3_ molecules, as noted above, state *B_A_* is preferred and PIP_3_ molecules are present at the CA, NCA, and third sites, and also more diffusely around the whole footprint of the bound PH domain. Thus, the PH domain can alternate between two orientations with different lipid footprints and the relative contribution of these two patterns of interaction dependent on the concentration of PIP_3_ molecules present in the membrane.

### Binding mechanism

**Fig. 5:**
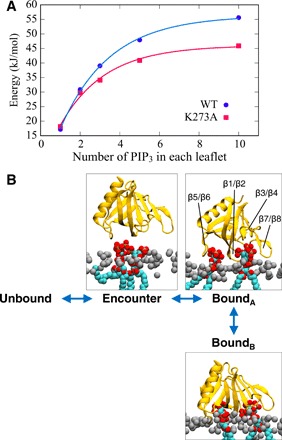
A mechanism of PH binding proceeding via an encounter state to two bound states. (**A**) Depth of minimum in the PMF (see also Fig. 1C) of the GRP1 PH domain as a function of the number of PIP_3_ molecules in each leaflet of the bilayer. Data points for the WT PH domain are shown for simulations using REUS and 1 to 10 PIP_3_ molecules in each leaflet of the bilayer (blue). The red points correspond to REUS simulations of the K273A mutant. (**B**) Schematic of a three-step mechanism for binding of the GRP1 PH domain to a bilayer containing multiple PIP_3_ molecules (see main text for details).

Experimental estimates of the dissociation constants for PIP_3_ from GRP1 PH range from 5 nM to 1 μM (*14*, *17*, *20*, *39*–*45*), which depend on the experimental conditions, e.g., pH condition, temperatures, and lipid bilayer composition. This corresponds to a free energy range of −48 to −34 kJ/mol. From the PMFs in Fig. 1, we can calculate dissociation constants (see Methods for details) and hence free energies of binding. For the 1, 2, 3, 5, and 10 PIP_3_ systems, this yields values for *K*_d_ (dissociation constant) = 2.1 × 10^−4^, 3 × 10^−6^, 1.6 × 10^−7^, 6.3 × 10^−9^, and 3.9 × 10^−10^ M, respectively, corresponding to free energies Δ*G* of −23, −34, −42, −51, and − 58 kJ/mol, respectively. Plotting the free energy minimum in PMFs (Fig. 5A for the REUS simulations and also fig. S4 for corresponding US simulations) suggests that there is no substantive increase in well depth beyond five PIP_3_ molecules present in the bilayer proximal leaflet to which the PH domain is bound. Examination of the 10 PIP_3_ systems (both REUS and US) suggested that, on average, ~4 PIP_3_ molecules were bound to the PH domain. Comparing the free energies of binding as a function of the number of PIP_3_ molecules present with the range of experimental estimates suggests that the most likely state of the PH domain is bound to between three and five PIP_3_ molecules.

A single mutation K273A within the canonical PIP binding site results in loss of experimentally detectable PIP_3_ binding to the GRP1 PH domain (*41*). Our simulations suggest that the protein-lipid interactions for the mutant are reduced by up to 10 kJ/mol, depending on the local concentration of PIP_3_. This difference between calculations and experiment may reflect the limitations of the current CG model (*46*) in describing the K273A mutant and/or may reflect the sensitivity of binding experiments to the conditions used. For example, while our simulations measured the interactions of the PH domain with a PIP_3_-containing lipid bilayer, the experiments on the K273A mutant (*41*) measured binding of the PH domain to either Ins(1,3,4,5)P_4_ (i.e., the water-soluble head group of PIP_3_) or to the corresponding di-C8-phosphoinositide in aqueous solution. The second bound state, *B_B_*, is not heavily populated for the K273A mutant in the presence of three PIP_3_ molecules (see fig. S3) in contrast to the wild-type (WT) PH domain under similar conditions (see above). This suggests the mutation perturbs both the strength and mode of interaction of the domain with a PIP_3_-containing membrane.

## DISCUSSION

Our simulations and PMF calculations indicate that a PH domain can bind simultaneously to multiple PIP molecules in a bilayer, via both the canonical and noncanonical sites alongside a third site and further less well spatially defined interactions. Interactions at these three binding sites increase the overall avidity of the PH domain for a PIP-containing membrane. This is seen in the dependence of the binding free energy (above), which indicates that assuming the GRP1 PH domain binds to at least three molecules of PIP_3_ gives good agreement with the range of measured dissociation constants for this interaction.

Inspection of the free energy landscape for this interaction suggests a three-stage mechanism for the interaction of the GRP1 PH domain with a PIP-containing membrane, proceeding via an initial *Encounter* state to two distinct bound states *B_A_* and *B_B_* that differ in the orientation of the PH domain and the depth of penetration of the bilayer (Fig. 5B). This may be compared with an earlier model of Lai *et al.* (*20*) who proposed that a transient membrane association state leads to the PH domain bound to PIP_3_, thereby enabling a two-dimensional search of the membrane surface for the target lipid. Our data suggest that this mechanism corresponds to a complex free energy landscape with two bound states, the relative occupancies of which is influenced by the (local) concentration of PIP_3_. Thus, the nature (strength and orientation) of the interaction of the PH domain with the bilayer surface will be influenced by nanoscale clustering of PIP_3_ and possibly of other molecules within the membrane. This correlates with previous studies of clustering of PIP molecules around bound PH domains (*30*). Our simulations should enable the design of further experiments to probe the relationship between nanoscale clustering of PIPs and local (i.e., single molecule) binding affinities of PH and related membrane recognition domains.

In addition to the influence of nanoscale clustering of PIP molecules, as noted above, the presence of anionic lipid species other than PIPs (e.g., PS) in a bilayer can influence the affinity of GRP1 PH for PIP_3_ (*17*). One can envisage competing effects of background anionic lipids on PH domain interactions with (multiple) PIP molecules in a membrane, namely, (i) a nonspecific electrostatic effect whereby an anionic membrane surface potential favors the formation of an initial encounter complex [cf. (*47*)] and (ii) a specific effect whereby binding of one or more anionic lipid headgroups to sites on the PH domain competes directly with PIP_3_ molecules for those sites. This latter effect might be expected to weaken the interaction of a PH domain with a PIP-containing membrane, relative to the interaction of the PH domain with multiple PIP molecules. Preliminary simulations suggest a complex interplay between the electrostatic environment presented by the membrane surface and the number of PIP_3_ molecules bound to the PH domain. A more systematic analysis of the effects of background anionic lipids on the free energy landscapes of the interaction of PH domains with a PIP-containing bilayer would be an appropriate subject for a future study. In the experimental literature, no significant GRP1 PH domain binding is seen for bilayers containing ~20% PS in the absence of PIP_3_ (*17*, *44*). In simulations, a PMF calculated for interaction of GRP1 PH with a bound PS molecule (unpublished data) did not reveal any greater interaction than that for the same domain interacting with a pure phosphatidylcholine (PC) bilayer. Thus, we may conclude that the binding of PIP_3_ to GRP1 PH cannot be substituted for by a high concentration of a simple anionic lipid such as PS.

Overall, our studies suggest that recognition of specific cell membranes by PH domain may be achieved by coincidence detection, either of locally clustered PIP molecules or of PIP molecules alongside other anionic lipids (*2*), the latter as suggested by data on the effects of other anionic lipids [e.g., (*7*, *17*)]. Thus, high-avidity interaction of a PH domain with a membrane would require a local nanoscale cluster of PIP molecules in an anionic lipid-enriched background. A quantitative mechanistic understanding of the nature of these interactions, for PH and for other membrane recognition domains, will be essential if we wish to intervene therapeutically in a rational fashion when correct membrane recognition is impaired by mutation or other disease processes.

## METHODS

### Simulations

To investigate of interactions of a membrane-bound protein on a membrane surface, we performed CG-MD simulations of the GRP1 PH domain interacting with a PIP_3_-containing lipid bilayer. For the structure of GRP1 PH domain, we used the crystal structure of the GRP1 PH domain bound to an Ins(1,3,4,5)P_4_ molecule (Protein Data Bank ID: 1FGY). A single mutation on the GRP1 PH domain (K273A) was modeled using MODELLER (*48*). The bilayer used in the simulations consisted of symmetric 1-palmitoyl-2-oleoyl-sn-glycero-3-phosphocholine (POPC)/PIP_3_ bilayers (234/2, 232/4, 230/6, 226/10, or 216/20 molecules). The systems were solvated with 6000 CG water molecules, and NaCl ions at a concentration of 150 mM were added to neutralize the system. The Martini 2.1 force field (*49*) was used for the CG model of the protein (residues 264 to 380, with a total charge of +3 for the resultant protein model), and the phosphates of PIP_3_ were assumed to be fully ionized, yielding a total change of −7 for the lipid headgroup). An elastic network model was applied to all backbone particles within a cutoff distance of 0.7 nm to model secondary and tertiary structure (*50*). The bond lengths were constrained to equilibrium lengths using the LINCS algorithm (*51*). Lennard-Jones and Coulombic interactions are cut off at 1.1 nm, with the potentials shifted to zero at the cutoff (*52*).

In the initial configuration of each simulation, the PH domain was displaced away from the lipid bilayer surface. All systems were subjected to steepest-descent energy minimized to remove the initial close contacts and equilibrated for 1 ns with the protein backbone particles restrained in NPT constant CG-MD simulations. A time step of 30 fs was used. The neighbor list was updated every 20 steps using the Verlet neighbor search algorithm. The systems were subject to pressure scaling to 1 bar using Parrinello-Rahman barostat (*53*), with temperature scaling to 323 K using velocity-rescaling method (*54*) with coupling times of 1.0 and 12.0 ps.

### Estimation of PMFs

The last window frame of the pre-equilibrated simulation was used for initial configuration for the unbound states. For the production runs of the REUS-MD simulation, the PLUMED2 package (version 2.3.3) (*55*) was used to patch GROMACS 5.1.4 (*56*), define the CVs, and perform the biasing. REUS-MD simulations were produced using a similar protocol used in a previous study for lipid interaction with transmembrane protein within lipid membranes (*36*). Replica exchanges were evaluated using the Boltzmann criterion. A CV was defined by a minimum distance between protein amino acids and phosphate group of lipids. US windows were set up with 16 windows, with the CV linearly spaced distances from 0.4 to 1.5 nm, with a force constant of 1000 kJ mol^−1^ nm^−2^. Exchanges of replicas were attempted every 1000 steps. The simulations were performed for 15 μs for each replica, yielding a total REUS-MD simulation time of 240 μs. For the analysis of each simulation, data for 0 to 2 μs were discarded before collecting data from 2 to 15 μs, which yield good convergence of the PMFs (see fig. S2). Only for the system of WT (10 PIP3), 20-μs simulation was performed for each replica. Multiple binding and unbinding transitions were observed in the continuous trajectories obtained by following the replicas. The unbiased PMFs after subtracting the effect of the umbrella potentials were calculated by the weighted histogram analysis method (*57*, *58*). The two-dimensional free energy surface for other variables, ξ_2_ and ξ_3_, was estimated (using locally written code) by reweighting the trajectories obtained by the REUS-MD simulation biasing along a single CV, ξ_1_ (*59*). Note that this assumes that the REUS-MD simulations for ξ_1_ provided sufficient sampling for the other variables, ξ_2_ and ξ_3_. US simulations were performed as described previously (*34*, *35*). Molecular graphics images were generated using VMD (*60*).

### Calculation of the density of lipid around the protein

The density of phosphate headgroups of PIP_3_ corresponding to each bound state was calculated with the unbiased distribution obtained from the REUS simulation. Each bound state was distinguished from the free energy surfaces. The bound state *B_A_* was defined as protein-lipid minimum distance [0.46, 0.52], protein-membrane COM distance [3.91, 3.97], and cosθ [0.36, 0.46]. The bound state *B_B_* was defined as protein-lipid minimum distance [0.45, 0.51], protein-membrane COM distance [3.45, 3.51], and cosθ [0.89, 0.99]. A cutoff distance of 0.7 nm was used for the protein-lipid contact, corresponding to a generally used definition for protein-lipid interactions for the MARTINI CG model.

### Calculation of the dissociation constant

For binding of a protein to a membrane in a periodic box with the membrane perpendicular to the *z* axis

$$K_{d}=[M]\frac{1-y}{y}=\frac{1}{N_{A}{\text{AL}}_{z}}\frac{\int_{b}^{L_{z}}\text{exp}[-\beta F(r)]\text{dr}}{\int_{0}^{b}\text{exp}[-\beta F(r)]\text{dr}}$$

where [*M*] is the molar ratio of the protein, *y* is the fraction bound, *A* is the *x*-*y* area of the membrane, *L_z_* is the box length in the *z* direction, *N*_A_ is Avogadro’s number, and *F*(*r*) is the PMF for association on the membrane. *F*(*r*) should be the constant zero above the bound distance *b*, and then we get

$$K_{d}=\frac{1}{N_{A}A}\frac{\left(1-b/L_{z}\right)}{\int_{0}^{b}\text{exp}[-\beta F(r)]\mathrm{dr}}$$

Taking the limit as *L_z_* → ∞, we get

$$\frac{1}{K_{d}}=N_{A}A\int_{0}^{b}\text{exp}[-\beta F(r)]\mathrm{dr}$$

Here, we used *b* = 1 nm.

## Supplementary Material

http://advances.sciencemag.org/cgi/content/full/6/8/eaay5736/DC1

Download PDF

Multiple lipid binding sites determine the affinity of PH domains for phosphoinositide-containing membranes

## SUPPLEMENTARY MATERIALS

Supplementary material for this article is available at http://advances.sciencemag.org/cgi/content/full/6/8/eaay5736/DC1 Fig. S1. PMFs for the GRP1 PH domain with a single mutation (K273A) interacting with lipid bilayers containing 1 to 10 PIP_3_ molecules. Fig. S2. Convergence of PMF calculations. Fig. S3. Free energy maps for the GRP1 PH domain with a single mutation (K273A) interacting with a lipid bilayer including 1 to 10 PIP_3_ molecules in each leaflet. Fig. S4. PMFs from US for the GRP1 PH domain interacting with lipid bilayers containing 1, 2, or 10 PIP_3_ molecules.

## Appendix

View/request a protocol for this paper from *Bio-protocol*.
