# Supplementary material for: Multiple lipid binding sites determine the affinity of PH domains for phosphoinositide-containing membranes
Source: Sci Adv. 2020 Feb 19;6(8):eaay5736. doi: 10.1126/sciadv.aay5736 (PMC7030919; doi:10.1126/sciadv.aay5736)
Supplement: http://advances.sciencemag.org/cgi/content/full/6/8/eaay5736/DC1 [file supp_6_8_eaay5736__index.html]

Science Advances | Science AdvancesAAASSearchScience AdvancesMenu

## Supplementary Materials

**This PDF file includes:**

- Fig. S1. PMFs for the GRP1 PH domain with a single mutation (K273A) interacting with lipid bilayers containing 1 to 10 PIP3 molecules.
- Fig. S2. Convergence of PMF calculations.
- Fig. S3. Free energy maps for the GRP1 PH domain with a single mutation (K273A) interacting with a lipid bilayer including 1 to 10 PIP3 molecules in each leaflet.
- Fig. S4. PMFs from US for the GRP1 PH domain interacting with lipid bilayers containing 1, 2, or 10 PIP3 molecules.

Download PDF

**Files in this Data Supplement:**

- Adobe PDF - aay5736\_SM.pdf
