# Supplementary material for: Multiple lipid binding sites determine the affinity of PH domains for phosphoinositide-containing membranes
Source: Sci Adv. 2020 Feb 19;6(8):eaay5736. doi: 10.1126/sciadv.aay5736 (PMC7030919; doi:10.1126/sciadv.aay5736)
Supplement: Download PDF [file aay5736_SM.pdf]

Science Advances
AAAS

advances.sciencemag.org/cgi/content/full/6/8/eaay5736/DC1

Supplementary Materials for

Multiple lipid binding sites determine the affinity of PH domains for phosphoinositide-containing membranes

Eiji Yamamoto*, Jan Domański, Fiona B. Naughton, Robert B. Best, Antreas C. Kalli, Phillip J. Stansfeld, Mark S. P. Sansom*

*Corresponding author. Email: eiji.yamamoto@sd.keio.ac.jp (E.Y.); mark.sansom@bioch.ox.ac.uk (M.S.P.S.)

Published 19 February 2020, *Sci. Adv.* **6**, eaay5736 (2020)
DOI: 10.1126/sciadv.aay5736

This PDF file includes:

Fig. S1. PMFs for the GRP1 PH domain with a single mutation (K273A) interacting with lipid bilayers containing 1 to 10 PIP_3 molecules.
Fig. S2. Convergence of PMF calculations.
Fig. S3. Free energy maps for the GRP1 PH domain with a single mutation (K273A) interacting with a lipid bilayer including 1 to 10 PIP_3 molecules in each leaflet.
Fig. S4. PMFs from US for the GRP1 PH domain interacting with lipid bilayers containing 1, 2, or 10 PIP_3 molecules.

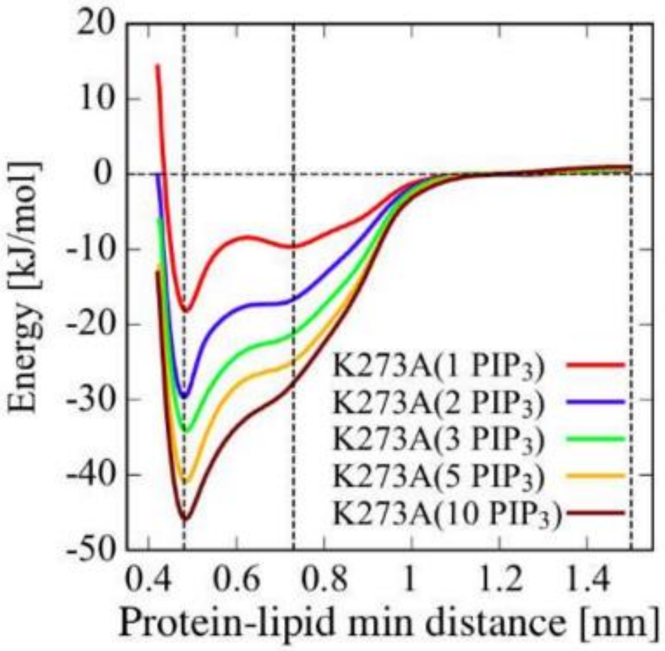

Fig. S1. PMFs for the GRP1 PH domain with a single mutation (K273A) interacting with lipid bilayers containing 1 to 10 PIP_3 molecules.

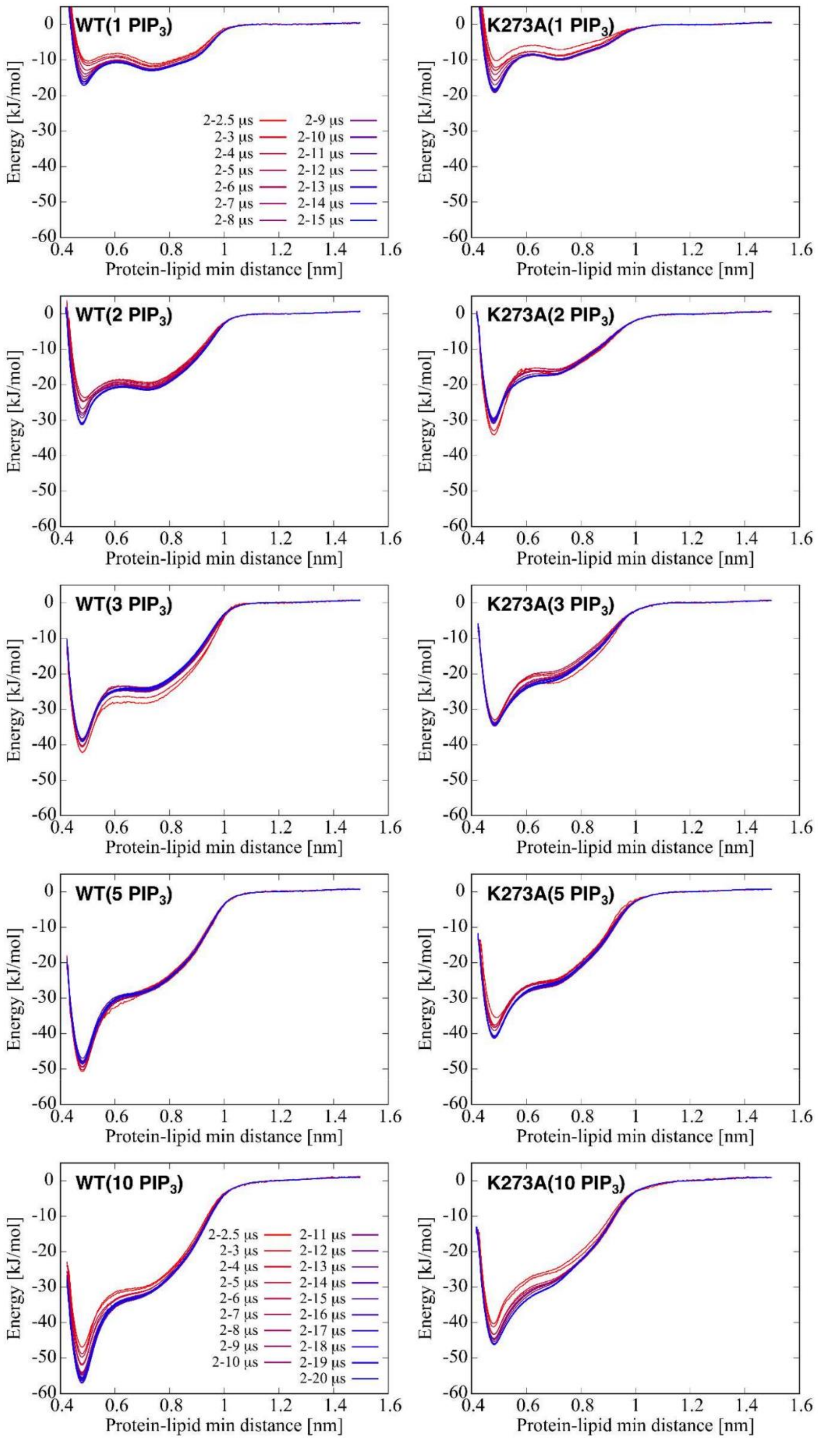

Fig. S2. Convergence of PMF calculations.

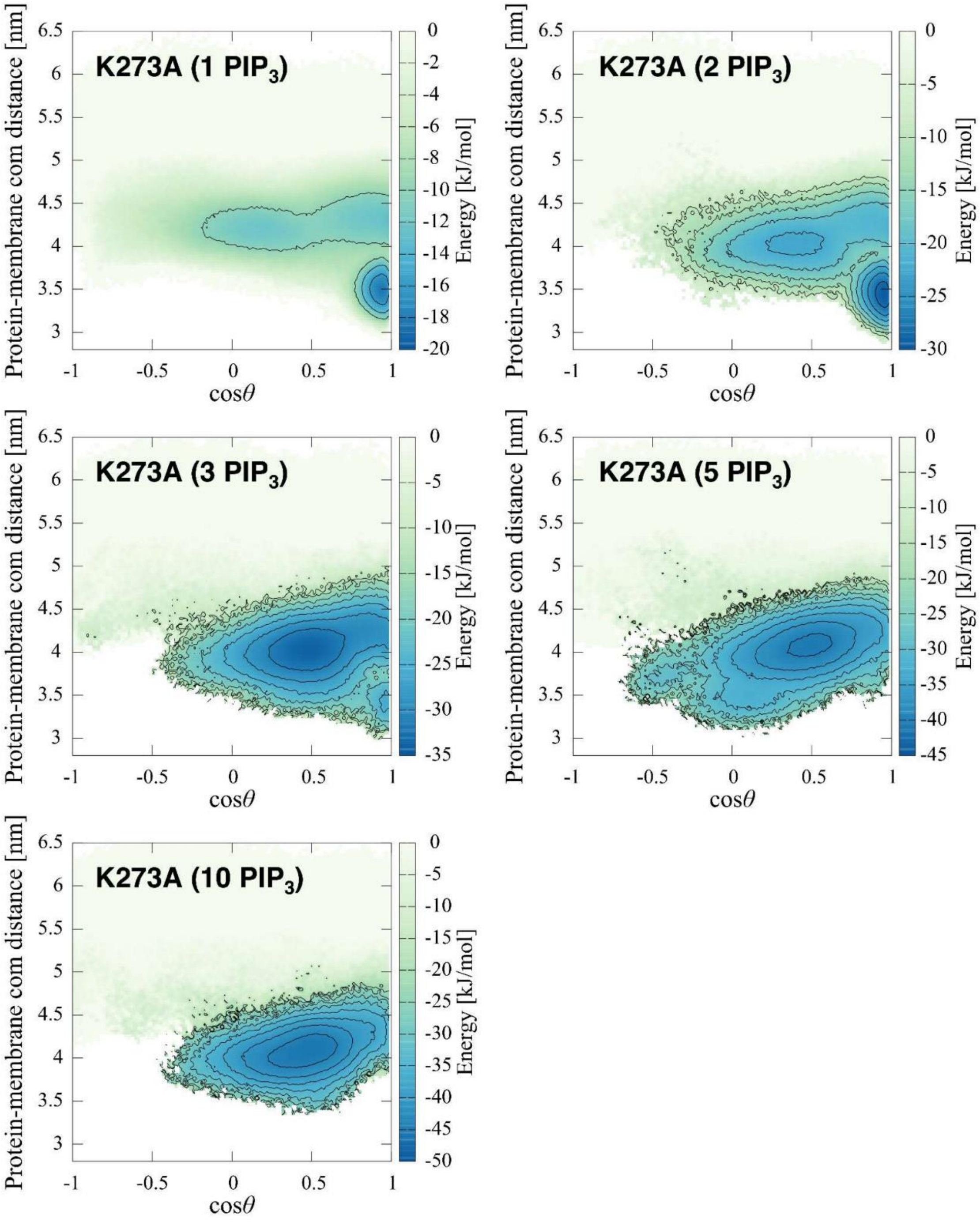

Fig. S3. Free energy maps for the GRP1 PH domain with a single mutation (K273A) interacting with a lipid bilayer including 1 to 10 PIP_3 molecules in each leaflet.

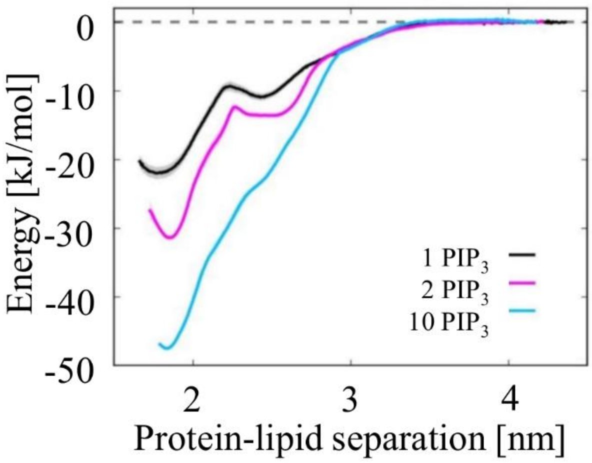

Fig. S4. PMFs from US for the GRP1 PH domain interacting with lipid bilayers containing 1, 2, or 10 PIP_3 molecules.
